# Discovering the link between IL12RB1 gene polymorphisms and tuberculosis susceptibility: a comprehensive meta-analysis

**DOI:** 10.3389/fpubh.2024.1249880

**Published:** 2024-01-22

**Authors:** Jie Huang, Qiurong He, Lijun Huang, Liping Liu, Pei Yang, Min Chen

**Affiliations:** ^1^Department of Clinical Laboratory, West China School of Public Health and West China Fourth Hospital, Sichuan University, Chengdu, China; ^2^Department of Clinical Laboratory, The First Affiliated Hospital of Hainan Medical University, Haikou, China

**Keywords:** IL12RB1, single-nucleotide polymorphism, tuberculosis, meta-analysis, susceptibility

## Abstract

**Introduction:**

Numerous studies suggest that the risk of tuberculosis (TB) is linked to gene polymorphisms of the interleukin-12 receptor b subunit 1 (IL12RB1), but the association between IL12RB1 polymorphisms and TB susceptibility has not been thoroughly investigated.

**Methods:**

A meta-analysis was conducted based on eight case-control studies with 10,112 individuals to further explore this topic. A systematic search of PubMed, Web of Science, Excerpt Medica Database, and Google Scholar up until April 6th, 2023 was performed. ORs and 95% CIs were pooled using the random-effect model. The epidemiological credibility of all significant associations was assessed using the Venice criteria and false-positive report probability (FPRP) analyses.

**Results:**

The IL12RB1 rs11575934 and rs401502 showed solid evidence of no significant association with TB susceptibility. However, a weak association was observed between the IL12RB1 rs375947 biomarker and pulmonary tuberculosis (PTB) susceptibility (OR = 1.64, 95% CI: 1.22, 2.21).

**Discussion:**

These findings should be confirmed through larger, better-designed studies to clarify the relationship between biomarkers in IL12RB1 gene and different types of TB susceptibility.

## 1 Introduction

Tuberculosis (TB), which is primarily caused by the bacterium *Mycobacterium tuberculosis*, is a severe infectious disease that can affect multiple organs, including the lungs, lymph nodes, and bones, among others (1). Despite advances in treatment and prevention, TB still represents a major cause of death around the world and poses a significant threat to global public health, particularly in developing nations (2). In 2021, the World Health Organization estimated more than 10 million people became ill with TB and 1.6 million deaths were related to the disease (1).

Approximately one in four individuals worldwide are infected with *M. tuberculosis*; however, only a small fraction (~10%) of these individuals ultimately develop active TB disease (3). Host genetics appear to have a significant impact on the likelihood of an individual’s susceptibility to mycobacterial infection and the subsequent development of TB (4). In recent decades, genetic research has identified germ-line mutations in fifteen genes, causing Mendelian susceptibility to mycobacterial disease (MSMD). The fifteen identifiable genes include: IL12B, IL12RB1, IL12RB2, IL23R, STAT1, IFNGR1, IFNGR2, IRF8, JAK1, SPPL2A, ISG15, TYK2, RORC, NEMO and CYBB (5). Among these genes, complete deficiency of interleukin-12 receptor b subunit 1 (IL12RB1) is the most common genetic etiology (5). The IL12RB1 gene plays a crucial role in regulating both innate and adaptive immunities (6). The gene encodes a subunit of the interleukin-12 receptor (IL-12R), which is essential for the production of interferon-gamma and the activation of Th1 cells (7). Deficiencies in IL12RB1 have been linked to increased susceptibility to tuberculosis and other intracellular infections (8). To date, 92 rare variants in the IL12RB1 gene have been reported in MSMD patients (Supplementary Table S1) (9–33). However, the majority of sporadic TB cases lack these rare mutations (4). An increasing number of studies have recently provided some support for the hypothesis that TB risk is associated with IL12RB1 polymorphisms. A single nucleotide polymorphism (SNP) is defined as a variation in a single nucleotide that occurs at a specific position in the genome. This variation is present in a significant portion of the population (typically more than 1%). SNPs can occur in coding (gene) regions, non-coding regions, or in the intergenic regions between genes. They are the most common type of genetic variation among people and can serve as biological markers, helping scientists locate genes associated with disease. Nevertheless, these studies have produced inconsistent results, and most of the findings could not be replicated in subsequent research. For instance, Kusuhara et al. linked the development of TB to three single nucleotide polymorphisms (SNPs) of IL12RB1 (i.e., rs11575934, rs375947, and rs401502) (34), whereas some other studies reported no such association (35, 36). It’s worth noting that the candidate-gene association studies published to date have small sample sizes, making it challenging to detect minor genetic effects (37). However, a meta-analysis of data from multiple studies can be helpful in increasing the statistical power to evaluate the coherence of an association.

Therefore, our study aimed to conduct a meta-analysis of the current literatures to summarize the knowledge of the relationship between the polymorphisms of IL12RB1 and TB susceptibility. This study can contribute to the development of biosignatures that aid in assessing the vulnerability of TB patients in the future.

## 2 Materials and methods

### 2.1 Literature search

The present study adhered to both the Human Genome Epidemiology Network (HuGENet) guidelines for systematic reviews of genetic association studies and the Preferred Reporting Items for Systematic Reviews and Meta-Analyses (PRISMA) statement.

A comprehensive search was conducted through various databases, namely PubMed, Web of Science, Excerpt Medica Database, and Google Scholar, using the core search terms (“Interleukin 12 Receptor Subunit Beta 1” OR “IL12RB1”) AND (“tuberculosis” OR “TB”). The search was limited to genetic association studies concerning tuberculosis published before April 6th, 2023. Additionally, a manual search was conducted on the reference lists of the relevant studies found in the original search to identify any further eligible studies.

### 2.2 Selection criteria

Our meta-analysis involved studies that met four pre-defined inclusion criteria: (1) they were either case–control or cohort studies that examined the correlation between mutations in IL12RB1 gene and the susceptibility to TB; (2) they provided either the odds ratios (ORs) and the corresponding 95% confidence intervals (CIs) for one or more models, or alternatively the allele frequencies that were necessary to calculate the ORs and CIs; (3) they were published in peer-reviewed English-language journals; (4) they were original research articles. Studies were excluded based on three criteria: (1) they were case reports, meta-analyses, or reviews; (2) they did not provide sufficient data; or (3) they included duplicated data (duplicated data: the data in different studies originate from the same cohort).

### 2.3 Data extraction and quality assessment

The study’s two researchers extracted data independently from the studies that were included. Data extracted included PubMed ID, author’s first name, year of publication, ethnicity of participants, source population, sample size, criteria for selecting study subjects, genotype distributions, allele frequencies, Hardy–Weinberg equilibrium (HWE), odds ratios (ORs) with 95% confidence intervals (CIs) among the control group and genotyping method used. Disagreements were resolved by discussing with the lead researcher. The methodological quality of the included studies was evaluated by utilizing the Newcastle-Ottawa Scale (NOS) (38).

### 2.4 Statistical analysis

Meta-analyses were done only for variants with at least three independent datasets and at least two for subgroup analyses. Statistical analyses were conducted using Stata software, version 13 (StataCorp, College Station, TX). Mutations with at least three independent datasets were included in the meta-analyses. Primary analyses employed an additive model for meta-analysis. If available, dominant and recessive models were also conducted to evaluate the associations between genetic mutations and TB risk. We also conducted subgroup analyses by ethnicities if sufficient data was available. ORs and 95% CIs were pooled using the random-effect model. The statistical significance threshold for all meta-analyses was set at *p* < 0.05, and all tests were conducted as two-tailed.

### 2.5 Assessment of cumulative evidence

Using the Venice criteria, we assessed the epidemiological credibility of all significant associations with tuberculosis susceptibility through meta-analyses and categorize them as strong, moderate, or weak based on the amount of evidence, replication, and protection from bias (39–41). The amount of evidence is graded based on the total number of test alleles or genotypes in both cases and controls, replication is graded by the I^2^ statistic, and protection from bias is evaluated with a series of bias tests, including tests for publication bias, small-study bias, and an excess of significant findings. Amount of evidence was graded by the sum of test alleles or genotypes among cases and controls: A for more than 1,000, B for 100 to 1,000, and C for less than 100. Replication was graded by the I^2^ statistic: A for I^2^ values less than 25%, B for I^2^ values between 25% and 50%, and C for I^2^ values greater than 50%. Protection from bias was mainly determined by sensitivity analyses and a series of bias tests including publication bias, small-study bias, and an excess of significant findings. Briefly, protection from bias was graded as A if there was no observable bias, or C if bias was evident. Therefore, the cumulative epidemiological evidence for significant associations was thought to be strong if all three grades were A, moderate if all three grades were A or B, and weak if any grade was C.

Moreover, we also performed false-positive report probability (FPRP) analyses to determine whether a significant association could be a false-positive finding. A prior probability of 0.05 was used to estimate FPRP values, and a cutoff value of 0.20 was adopted. If the FPRP values were less than 0.05, we upgraded the cumulative evidence from weak to moderate or from moderate to strong. Conversely, if the FPRP values were greater than 0.20, we downgraded the cumulative evidence from strong to moderate or from moderate to weak.

Overall, by using the Venice criteria and FPRP analyses, we comprehensively evaluated the credibility of significant associations with tuberculosis susceptibility and provide a more accurate assessment of their potential significance.

### 2.6 Functional annotation

We identified all SNPs in linkage disequilibrium (LD) with the SNPs (i.e., r^2^ > 0.8) showing a significant association with risk of tuberculosis in our meta-analyses using data from the 1,000 Genomes Project. We evaluated the potential functional effect of nonsynonymous SNPs using the prediction algorithms LRT, MutationTaster, MutationAssessor and PolyPhen-2. HaploReg 4.2 is used for the noncoding variants’ the potential functional effect evaluating. We also delved into genome-wide eQTL data across various tissues, sourced from the Genotype-Tissue Expression (GTEx) Project and the Multiple Tissue Human Expression Resource Project. eQTLs, chromosomal regions influencing mRNA or protein expression levels, act as intermediaries connecting genetic mutations with phenotypes. This analysis offers novel insights into how mutations correlate with phenotypic manifestations.

## 3 Results

### 3.1 Literature search

**Figure 1:**
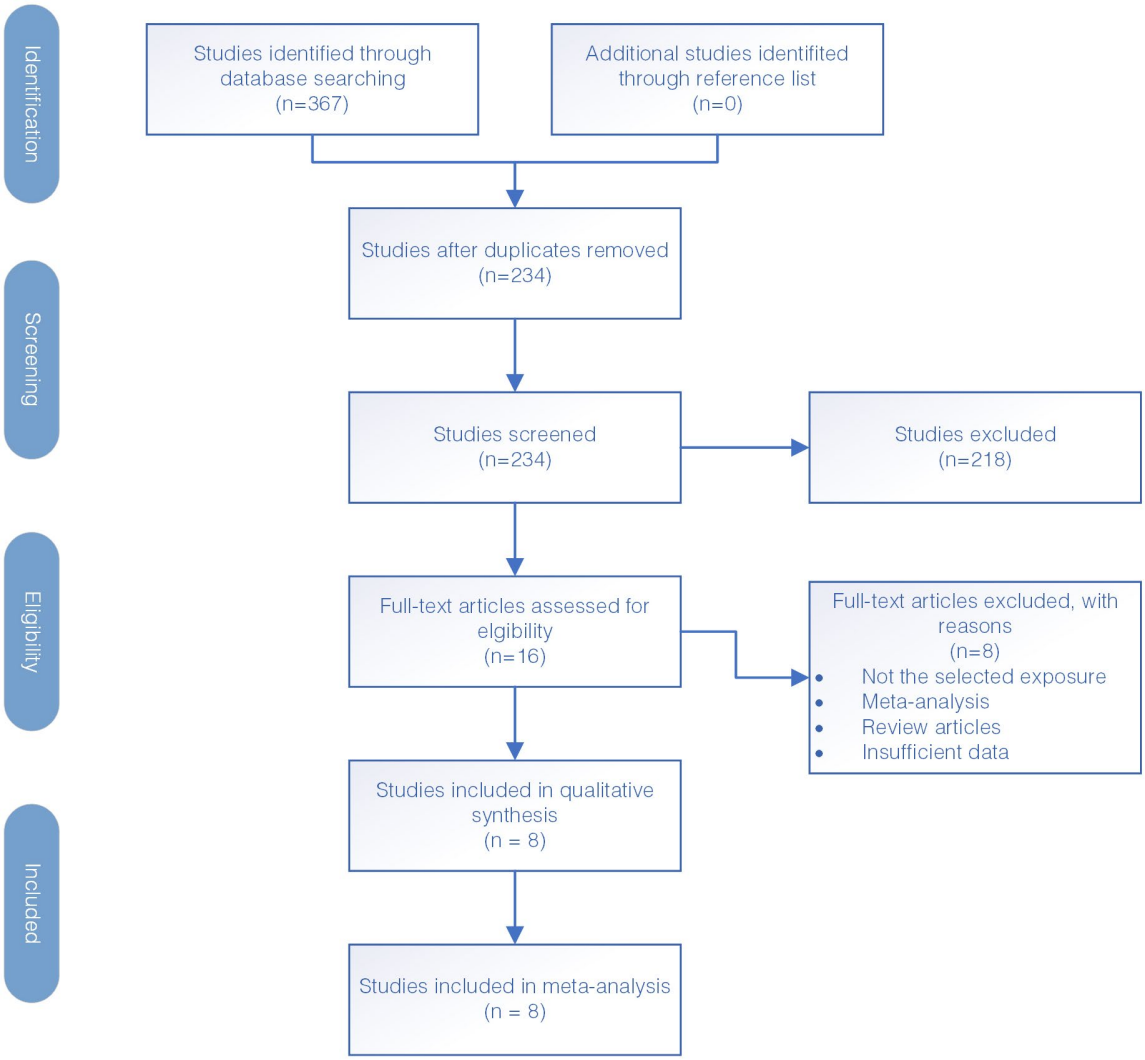
Flow diagram for identification of eligible studies for this meta-analysis.

The diagram illustrating the selection of studies is presented in Figure 1. Firstly, a total of 367 studies were identified through electronic database search. Out of all the studies found, 133 studies were identified as duplicates and removed, resulting in 234 studies that were screened further. During this screening process, we assessed the relevance of the studies to our research objectives by reviewing their titles and abstracts. Subsequently, 218 studies were excluded as they were deemed not relevant to the study’s purpose which included studies that do not focus on the IL12RB1 gene, studies not related to tuberculosis susceptibility, and those with insufficient genetic data necessary for our meta-analysis. The studies that met the inclusion criteria underwent a full-text screening for further evaluation. After thorough screening, eight case–control studies were deemed eligible for inclusion in the meta-analysis. The reference for these studies is provided as Kusuhara et al. (34), Zhang et al. (35, 36), Akahoshi et al. (42, 43), Lee et al. (44), Sahiratmadja et al. (45), and Meyer et al. (46).

### 3.2 Characteristics of included studies

The details of 8 included studies are presented in Table 1. Across all studies, a total of 10,112 participants were included with 4,843 patients with TB and 5,269 healthy controls. The average number of cases and controls across the studies was 605 (range: 87–1999) and 659 (range: 115–2,589), respectively. Of these studies, 5 only evaluated pulmonary TB while the remaining 3 evaluated both pulmonary and extra-pulmonary TB. Six studies were from East Asia, one from Southeast Asia, and one from Africa. The NOS score across all studies was shown in Table 1, with further details available in Supplementary Table S2. Meta-analyses were performed on five polymorphisms (rs11575934, rs401502, rs375947, rs11575935, and rs17852635) in IL12RB1, at least three datasets.

**Table 1:** Basic information of studies included in this meta-analysis.

PMID	First author	Publishing year	Source population	Ethnicity	Sample size (case/control)	Genotyping method	HWE	Diagnosed method of case	Type	NOS Quality score	Reference
12596048	Mitsuteru	2003	Japan	East Asian	98/197	PCR-RFLP	Yes	Sputum smear	PTB	8	(42)
15004750	Mitsuteru	2004	Japan	East Asian	114/115	PCR	Yes	Sputum smear	PTB	8	(43)
16088278	Hye	2005	Korea	East Asian	115/151	PCR-RFLP	Yes	Bacteriologically or pathologically confirmed	TB	7	(44)
17284226	Kusuhara	2007	Japan	East Asian	87/265	PCR	Yes	Culture or smear or PCR	PTB	6	(34)
17392024	Edhyana	2007	Indonesia	Southeast Asian	382/437	PCR	Yes	Clinical symptoms, CXR, culture, smear	PTB	8	(45)
25360588	Zhang	2014	China	East Asian	1032/1008	MassArray	Yes	Smear or culture, clinical–radiological and histological diagnosed	TB	7	(35)
26242990	Christian	2016	Ghana	African	1999/2589	PCR	Yes	Culture or smear	PTB	8	(46)
32349793	Zhang	2020	China	East Asian	1016/507	MassArray	Yes	Smear or culture, radiological and histological diagnosed	TB	7	(36)

PMID, PubMed ID; PCR, polymerase chain reaction; PCR-RFLP, PCR-restriction fragment length polymorphism; HWE, Hardy–Weinberg equilibrium; CXR, Chest X-ray; TB, tuberculosis; PTB, pulmonary tuberculosis; NOS, the Newcastle-Ottawa scale; SNPs, single nucleotide polymorphisms.

### 3.3 Association of IL12RB1 rs11575934 polymorphism with TB susceptibility

Totally, 8 case–control studies (34–36, 42–46) (4,843 cases and 5,269 controls) were included in the meta-analysis investigating the IL12RB1 rs11575934 polymorphism. There was no significant association in the additive model (OR = 1.04, 95% CI 0.91–1.19), the dominant model (OR = 1.00, 95% CI 0.83–1.22), and the recessive model (OR = 1.04, 95% CI 0.75–1.45). Also, the association of IL12RB1 rs11575934 polymorphism with TB risk were not found in subgroup-analyses (PTB and East Asian subgroup) (Table 2).

**Table 2:** Results of association between IL12RB1 gene polymorphisms and TB susceptibility.

Polymorphisms	Genetic models	Subgroup	*n*	OR (95%CI)	*p*	*I*^2^ (%)
rs11575934	Additive model					
		Total	8	1.04 (0.91, 1.19)	0.051	56.5
		PTB	5	1.18 (1.00,1.40)	0.158	30.2
		East Asian	6	1.05 (0.86, 1.28)	0.625	65.8
	Dominant model					
		Total	4	1.00 (0.83, 1.22)	0.980	49.8
		PTB	2	1.27 (0.79, 2.05)	0.328	58.3
		East Asian	3	1.01 (0.78,1.32)	0.928	65.4
	Recessive model					
		Total	4	1.04 (0.75, 1.45)	0.806	64.2
		PTB	2	1.38 (0.67, 2.83)	0.390	71.5
		East Asian	3	1.09 (0.70, 1.69)	0.719	76.1
rs401502	Additive model					
		Total	7	1.06 (0.92, 1.22)	0.459	59.3
		PTB	4	1.34 (0.96, 1.88)	0.085	61.1
		East Asian	5	1.07 (0.87, 1.32)	0.509	70.9
	Dominant model					
		Total	3	1.08 (0.82, 1.41)	0.601	67.6
		East Asian	3	1.08 (0.82, 1.41)	0.601	67.6
	Recessive model					
		Total	3	1.11 (0.72, 1.71)	0.634	75.7
		East Asian	3	1.11 (0.72, 1.71)	0.634	75.7
rs375947	Additive model					
		Total	5	1.07 (0.87, 1.32)	0.538	71.2
		**PTB**	**2**	**1.64 (1.22, 2.21)**	**0.001**	**0**
		East Asian	5	1.07 (0.87, 1.32)	0.538	71.2
	Dominant model					
		Total	3	1.07 (0.82, 1.41)	0.619	67.8
		East Asian	3	1.07 (0.82, 1.41)	0.619	67.8
	Recessive model					
		Total	3	1.09 (0.70, 1.69)	0.700	76.6
		East Asian	3	1.09 (0.70, 1.69)	0.700	76.6
rs11575935	Additive model					
		Total	3	1.17 (0.65, 2.11)	0.605	44.6
		East Asian	2	1.40 (0.55, 3.54)	0.481	55.9
	Dominant model					
		Total	2	1.33 (0.51, 3.43)	0.561	71.4
	Recessive model					
		Total	2	1.55 (0.23, 10.60)	0.658	0
rs17852635	Additive model					
		Total	3	0.95 (0.80, 1.13)	0.556	53.8
		East Asian	3	0.95 (0.80, 1.13)	0.556	53.8
	Dominant model					
		Total	2	0.92 (0.80, 1.05)	0.205	0
		East Asian	2	0.92 (0.80, 1.05)	0.205	0
	Recessive model					
		Total	2	0.83 (0.62, 1.10)	0.187	43.2
		East Asian	2	0.83 (0.62, 1.10)	0.187	43.2

OR: odds ratio; CI: confidence interval. Bold values in the table indicate results with significant differences.

### 3.4 Association of IL12RB1 rs401502 polymorphism with TB susceptibility

Based on the findings of the meta-analysis of seven case–control studies (34–36, 42, 44–46), including 4,729 cases and 5,154 controls, no significant association was found between the rs401502 polymorphism and TB risk in the additive (OR = 1.06, 95% CI 0.92–1.22), dominant (OR = 1.08, 95% CI 0.82–1.41), and recessive (OR = 1.11, 95% CI 0.72–1.71) models. Additionally, no significant association was found in the East Asian subgroup, as shown in Table 2.

### 3.5 Association of IL12RB1 rs375947 polymorphism with TB susceptibility

**Figure 2:**
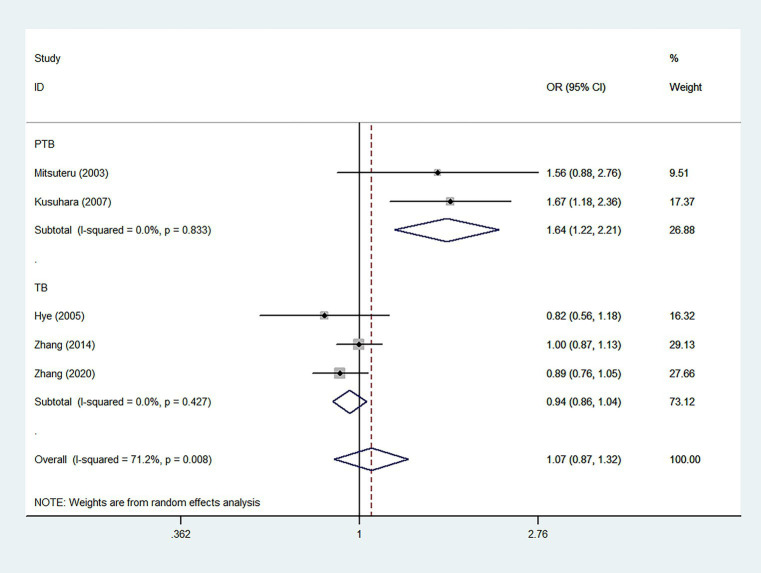
Forest plot of association between IL12RB1 rs375947 polymorphism and tuberculosis susceptibility in the additive model.

According to five case–control studies (34–36, 42, 44) (2,348 cases and 2,128 controls), the pooled ORs found no significant association between the IL12RB1 rs375947 polymorphism and susceptibility to TB, as shown in Table 2. Interestingly, in the subgroup analysis, the correlation between the IL12RB1 rs375947 polymorphism and susceptibility to pulmonary TB (PTB) was significant in the additive model (OR = 1.64, 95% CI: 1.22, 2.21), as shown in Table 2 and Figure 2.

### 3.6 Association of IL12RB1 rs11575935 and rs17852635 polymorphism with TB susceptibility

Three case–control studies (584/2,146 cases and 853/1712 controls) were included in the meta-analysis on the IL12RB1 rs11575935 and rs17852635, respectively. Based on our observations, we did not find any significant correlation between the two polymorphisms and susceptibility to TB, either in the observed models or subgroup analyses (Table 2).

### 3.7 Assessment of cumulative evidence

Our studies revealed a significant link between the IL12RB1 rs375947 polymorphism and the risk of PTB. This association received a grading of C, A, C across three categories—amount of evidence, replication, and protection from bias. Additionally, the FPRP value for the association was calculated as 0.073. Despite the statistical significance, we assigned a weak grade to the epidemiological credibility of this association. The details of assessment of cumulative evidence were seen in Supplementary Table S3.

### 3.8 Functional annotation

Rs375947 which was associated with PTB susceptibility is a missense change in exon 10 of IL12RB1. Algorithms developed to predict the effect of missense changes on protein structure and function (LRT, MutationTaster, MutationAssessor, Polyphen2) all suggest that this variant is likely to be tolerated (Supplementary Table S4A). Nineteen SNPs were identified strong linked (r^2^>0.80) with rs375947 according to the data form the 1,000 Genomes Project (Supplementary Table S4B). Most of them map to intronic region of the IL12RB1 and only three SNPs are in coding region of IL12RB1. According to data from HaploReg, rs372889, rs200396788, rs201422056, rs7255688, rs401502, rs391410, rs381607, rs365179 and rs447171 might be located in a region with strong enhancer activity; rs372889, rs391410, rs381607 and rs365179 might be located in a region with DNAse I hypersensitivity site. The GTEx Project shows that the rs375947 is eQTL for the IL12RB1, MAST3 and LRRC25 and associated with a decrease in these three genes (Supplementary Table S4C).

## 4 Discussion

Our study firstly assessed the link between IL12RB1 polymorphisms and TB susceptibility through a comprehensive meta-analysis. Overall, our findings indicated a weak epidemiological association between the IL12RB1 rs375947 polymorphism and PTB susceptibility. In contrast, we found solid evidence indicating no significant association between rs11575934 and rs401502 polymorphisms and the risk of TB. These findings are based on sufficient sample sizes of cases and controls, approximately 10,000 for each polymorphism.

The critical role of IL12RB1 in controlling mycobacterial infections was first highlighted by Altare et al. (47) and de Jong et al. (33), who reported cases of individuals with complete deficiency of IL12RB1 suffered from disseminated *Mycobacterium bovis* bacillus Calmette-Guerin (BCG) infections. IL12RB1 deficiency impairs the production of IFN-γ, which has been reported to be critical in controlling mycobacterial infection (6). Subsequently, this association has been substantiated across various ethnic groups, indicating that the effects of IL12RB1 on the dissemination of mycobacterial infections are almost omnipresent. For rare mutations, our study summarized 92 host disease-causing germline variants associated with mycobacterial infections from 271 published cases (Supplementary Table S1). Among these variants, the top five pathogenic mutations were c.1791 + 2 T > G, c.783 + 1G > A, p.S321*, p.G542*, and p.R173W. Most of these pathogenic variants are truncating variants (76%), while 24% are missense variants. These pathogenic variants contribute to the risk of familial syndromes with mycobacterial infections and TB. However, gene polymorphisms are more prevalent than rare mutations in most sporadic TB cases. Regarding the polymorphisms, we firstly conducted the meta-analysis that systematically evaluated the correlation between the IL12RB1 polymorphisms and TB susceptibility by analyzing eight case–control studies involving 10,112 individuals. Our meta-analyses found that the IL12RB1 rs375947 was associated with PTB risk. In addition to tuberculosis susceptibility, research has also found that this locus is associated with susceptibility to ER-negative tumors and atopic dermatitis (48, 49). However, this association was only observed in the PTB subgroup and not in the group that included both pulmonary and extrapulmonary tuberculosis. Different clinical phenotypes of tuberculosis susceptibility may be associated with different genes. To identify genes that are distinctly correlated, it is imperative to categorize various phenotypes in the research design. This emphasizes the need to choose a better-specified clinical phenotype while discussing the relationship between diseases and variants. A similar point was made by Abel et al. (4). Nevertheless, we assigned a weak-grade epidemiological credibility to this significant association, mainly due to the limited sample size. Therefore, more studies are required to clarify the association between the IL12RB1 rs375947 polymorphism and PTB susceptibility. Only 24 polymorphisms in IL12RB1 have been discussed for their association with TB susceptibility so far (34–36, 42–46). Therefore, more mutations in IL12RB1 needed to be detected to enrich knowledge of the association of IL12RB1 and TB susceptibility.

Nevertheless, certain limitations must be acknowledged. Firstly, the number of included studies was relatively limited. Secondly, substantial heterogeneity existed among studies. This heterogeneity may be attributed to various factors, such as the source of participants, inclusion criteria of case and control, level of exposition and diagnosis methods. Thirdly, TB infection could be facilitated by environmental factors like socioeconomic status, smoking/drinking history, and dietary habits, for which insufficient data were available for analysis.

In summary, this meta-analysis concluded that rs11575934 and rs401502 are not significantly associated with TB susceptibility, with a sample size of around 10,000 cases and controls, providing solid evidence for this claim. The IL12RB1 rs375947 polymorphism, on the other hand, was associated with PTB susceptibility. However, these findings should be confirmed through larger studies involving the subdivision of phenotypes to clarify the relationship between biomarkers in IL12RB1 gene and TB susceptibility.

## Data availability statement

The original contributions presented in the study are included in the article/Supplementary material, further inquiries can be directed to the corresponding authors.

## Author contributions

JH and QH contributed to the literature inclusion, data extraction, the data analysis, and the drafting and completion of the manuscript. LL and LH contributed to the data checking. MC and PY contributed to the study design and conception of the manuscript. JH, QH, LH, LL, PY, and MC reviewed the manuscript. All authors contributed to the article and approved the submitted version.
